# Development of a Physiologically Based Pharmacokinetic Model for Nitrofurantoin in Rabbits, Rats, and Humans

**DOI:** 10.3390/pharmaceutics15092199

**Published:** 2023-08-25

**Authors:** Raju Prasad Sharma, Elsje J. Burgers, Joost B. Beltman

**Affiliations:** Division of Drug Discovery and Safety, Leiden Academic Centre for Drug Research, Leiden University, Einsteinweg 55, 2333 CC Leiden, The Netherlands; rpsharmasysbio@gmail.com (R.P.S.); e.j.burgers@lacdr.leidenuniv.nl (E.J.B.)

**Keywords:** physiologically based pharmacokinetic model, renal insufficiency, drug-induced liver injury, nitrofurantoin

## Abstract

Nitrofurantoin (NFT) is a commonly used antibiotic for the treatment of urinary tract infections that can cause liver toxicity. Despite reports of hepatic adverse events associated with NFT exposure, there is still limited understanding of the interplay between NFT exposure, its disposition, and the risk of developing liver toxicity. In this study, we aim to develop a physiologically based pharmacokinetic (PBPK) model for NFT in three different species (rabbits, rats, and humans) that can be used as a standard tool for predicting drug-induced liver injury (DILI). We created several versions of the PBPK model using previously published kinetics data from rabbits, and integrated enterohepatic recirculation (EHR) using rat data. Our model showed that active tubular secretion and reabsorption in the kidney are critical in explaining the non-linear renal clearance and urine kinetics of NFT. We subsequently extrapolated the PBPK model to humans. Adapting the physiology to humans led to predictions consistent with human kinetics data, considering a low amount of NFT to be excreted into bile. Model simulations predicted that the liver of individuals with a moderate-to-severe glomerular filtration rate (GFR) is exposed to two-to-three-fold higher concentrations of NFT than individuals with a normal GFR, which coincided with a substantial reduction in the NFT urinary concentration. In conclusion, people with renal insufficiency may be at a higher risk of developing DILI due to NFT exposure, while at the same time having a suboptimal therapeutic effect with a high risk of drug resistance. Our PBPK model can in the future be used to predict NFT kinetics in individual patients on the basis of characteristics like age and GFR.

## 1. Introduction

Nitrofurantoin (NFT) is a commonly used oral antibiotic for treating lower urinary tract infections (UTIs) due to its broad-spectrum activity and low resistance among pathogens. It is used in the short term to treat acute urinary tract infections and in the long term as prophylaxis against recurrent infections. Unfortunately, it is also associated with drug-induced liver injury (DILI), which is the most common adverse effect of NFT exposure [1,2,3,4,5,6]. Severe fatalities due to hepatic toxicity have been reported [7,8], and autoimmune-like hepatitis has been identified as the most common cause [9,10]. Although the mechanisms by which NFT causes DILI are not fully resolved, in vitro studies have suggested that NFT may cause liver injury by hyper-activation of several cellular stress pathways [11].

While several hepatic adverse events associated with NFT exposure have been reported, there is still a large gap in understanding the relationship between NFT exposure, its disposition, and the occurrence of adverse events [12]. Individual factors, such as gender, advanced age, dosage regimen, and renal insufficiency, have been identified as increasing the risk of developing DILI, as well as the ineffectiveness of therapy [1,2,13,14,15,16]. Despite being on the market for almost 60 years, there are surprisingly few pharmacokinetics (PK) studies of NFT. Moreover, a concrete scientific basis to achieve optimal dosing of NFT is missing, especially for application to pediatric and geriatric subpopulations. For example, Muller et al. (2017) reported in a meta-analysis that low doses of NFT can be as effective as high doses when used long term [2]. Understanding the drug’s disposition inside the body represents an important first step toward understanding NFT-induced liver injury.

Nitrofurantoin has a short half-life of 2–3 h, with most of the drug eliminated through urine clearance, representing 25–40% of the administered dose [3]. The concentration of NFT in the urinary bladder, the main site of NFT’s antibacterial activity, determines its therapeutic effect. A study has shown that a minimum of 30% of the administered dose needs to reach the urinary bladder to obtain an optimal therapeutic effect. Animal studies have shown that the pharmacokinetics of NFT exhibits dose-dependent and non-linear kinetics, especially in the urine [17,18,19]. Additionally, human studies have shown that urine PK and plasma PK are highly variable between subjects [4]. Excretion of NFT in the urine of rats has been reported to be age dependent, and the gene ABCG2 (which codes for breast cancer resistance protein (BCRP), an efflux transporter) could play a major role in determining the NFT concentration in the urine [20,21,22,23]. Thus, the PK profile of nitrofurantoin is complicated, can be influenced by several factors, and is difficult to predict.

Physiologically based pharmacokinetic (PBPK) models have been useful to predict drug disposition in various organs, taking into account physiological information and drug properties. In this study, we aimed to develop a PBPK model for NFT in rabbits, rats, and humans using cross-species extrapolation. Our multi-species modeling approach allowed to integrate data acquired for various organs in either rats or rabbits, providing valuable mechanistic information to subsequently predict nitrofurantoin kinetics in humans. We evaluated the role of non-linear renal clearance in NFT drug disposition and the effect of gender, age, and renal function on this disposition in order to understand how these factors contribute to the efficacy and safety of NFT. We developed the model following a cross-species extrapolation approach, using rabbit experimental data to describe renal clearance, and extended with enterohepatic recirculation (EHR) on the basis of rat data. Our model showed that both active secretion and tubular reabsorption in the kidney are needed to explain the non-linear urine kinetics of NFT. The extrapolated human model successfully explained human NFT kinetics. Finally, we used the human PBPK model to explore how the glomerular filtration rate (GFR) of an individual is expected to impact NFT disposition and consequently the risk for NFT-induced liver injury and its therapeutic efficacy.

## 2. Methods

### 2.1. PBPK Model Structure to Describe NFT Kinetics

**Figure 1:**
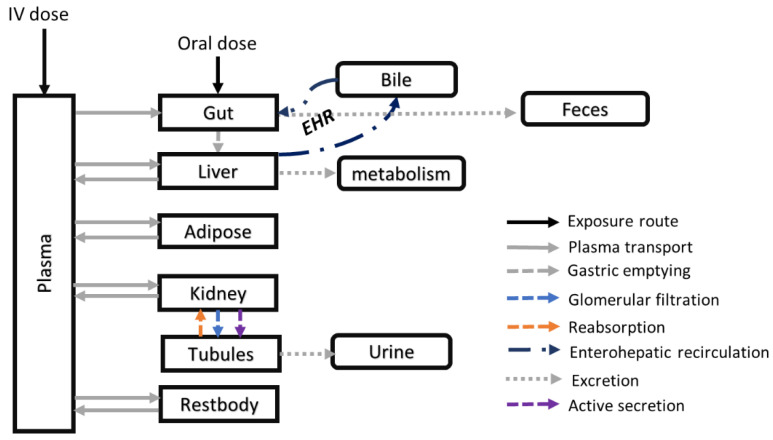
PBPK model structure to describe NFT kinetics. The model comprises 10 compartments, with each compartment representing a specific organ or tissue, except for the metabolism compartment, which represents the hepatic NFT clearance through metabolism. Organs that are not explicitly included in the model are represented by the ‘Restbody’ compartment. The model shows the blood flow to and from the organs, represented by solid arrows. The liver compartment can excrete the drug into bile, which can be reabsorbed from the gut through enterohepatic recirculation (EHR). The kidney, liver, and gut compartments can all clear NFT. NFT can be transported from the kidney to tubules through glomerular filtration and active secretion, as indicated by the dotted blue and violet arrows, respectively. The orange dotted arrow represents the reabsorption of NFT from tubules back into the kidney.

We developed a PBPK model to describe NFT kinetics in relevant organs of three mammal species, i.e., rabbit, rat, and human. We kept a consistent model structure across all species, including the following tissues: gut, liver, fat, kidney, plasma, and ‘rest of the body’ (Restbody), with physiological blood flow between these compartments (Figure 1). The gut compartment was further divided into two sub-compartments: gut and gut lumen. The kidney compartment included a filtrate sub-compartment, which received NFT from the kidney via species-specific GFRs. The filtrate then entered a ‘delay’ compartment, which acted as a temporary storage compartment from which NFT was excreted to urine via a first-order rate constant. The rest of the body represented all other tissues not explicitly included in the model, and its volume was derived by subtracting the volumes of all other compartments from the total body volume. We derived the blood flow to the rest of the body by subtracting the blood flows of all compartments entering venous blood from the cardiac output.

The PBPK model incorporated both intravenous (IV) and oral exposure routes. In the oral exposure route, the drug was administered directly to the gut compartment, entering the gut lumen at a constant rate. From the gut lumen, NFT entered the gut via first-order rate absorption and was then transported to the liver via the portal vein. Unabsorbed drug in the gut was excreted to feces via a first-order rate constant. IV doses of NFT enter directly the systemic circulation.

To account for NFT excretion in bile, an additional biliary compartment and an enterohepatic recirculation (EHR) process were included in the model. We described the unbound NFT that was available for distribution by multiplying the fraction unbound drug by the total NFT plasma concentration.

### 2.2. Modeling Strategy

First, we collected physiological data from the published literature to inform the PBPK model, including organ volumes and blood flow in rabbits, rats, and humans. We used perfusion-rate-limited kinetics to describe NFT tissue distribution and modeled liver metabolism with Michaelis–Menten kinetics. Based on the afore-presented model structure, we created four different versions of the model (model V1 to model V4), each with a different renal clearance mechanism, as described in the Results section. For parameter calibration (details later), we used kinetics data of plasma and urine measured in rabbits after IV administration at different doses. We used the Berezhkovskiy method to calculate the tissue:plasma partition coefficient for each compartment [24], except for the Restbody:plasma partition coefficient, which we calibrated on the basis of the data. We also fitted the hepatic clearance and renal clearance parameters.

We then extended the model by including enterohepatic recirculation (EHR), creating model V5. We modeled NFT efflux from the liver into bile using the Michaelis–Menten equation, considering this process to have a maximal efflux rate. We modeled the transfer of NFT from bile to the gut lumen with first-order rate kinetics. To parameterize this process, we used published rat kinetics data. Here, we kept the Restbody partition coefficient parameter values the same and scaled the renal clearance parameters (as estimated for model V4) based on rat body weight. We also created a subversion of model V5 (model V5a), where we used a hierarchical approach to fit species-specific EHR-related parameters while keeping the other parameters the same as in model V4. Finally, we extrapolated Model V5a to predict NFT kinetics in humans using allometric scaling [25]. Full model equations are available in the Supplementary Materials. Model parameters, including tissue volumes and blood flows, body weight, cardiac output, and drug-specific parameters, are also in the Supplementary Materials (Supplementary Tables S1–S4). We used published equations to include the dependence of such physiological parameters (body weight, cardiac output, organ volumes, and blood flows) on age [26].

### 2.3. Model Parameterization and Evaluation

To estimate model parameters, we used the Markov Chain Monte Carlo (MCMC) approach, as implemented in MCSim (ver. 6.1) [27], with input from experimental data collected from various literature sources (see later). We considered priors for parameters to be distributed either according to a normal distribution or a truncated normal distribution. The likelihood of the data was considered to follow a normal distribution with a coefficient of variation of 10%. Using a likelihood function, during model fitting, the parameter values were updated to result in posterior distributions. From these posteriors, we randomly selected 1000 parameter sets, created model simulations with each set, and calculated the 2.5 percentile, median, and 97.5 percentile simulated value for each time point. We compared the model-predicted plasma concentration curves with the observed plasma concentrations from literature data. To extract the plasma concentrations from the literature, we used WebPlotDigitizer (https://automeris.io/WebPlotDigitizer/ (accessed on 1 November 2022)). Plasma and urine kinetics data upon single oral or IV dosing of 0.25, 1.25, 2.5, 5, 10, and 15 mg/kg to the rabbits were extracted from [17]. Rat kinetics data upon IV or oral dosing were taken from [23,28]. We extracted human time course plasma data upon a single oral administration of three different doses (50, 100, and 200 mg) a day from [4]. Our strategy for model development and validation based on the independent data sets from the three species involved the following steps: First, we calibrated the model using IV dose kinetics data from rabbits. Second, we tested the same model using intravenous kinetics data from rats (referred to as model V4). Third, the final version of the model (referred to as model V5a) was tested on the basis of separate human data following oral dosing using cross-species extrapolation. Note that additional data to validate model predictions in multiple tissues of rats, rabbits, and humans would be highly beneficial, ideally involving measurements in plasma, urine, liver, and bile within the same individuals and within a single species.

We visually compared the model predictions with the experimental data and also investigated the effect of parameter variation on model output. For this purpose, we used a global sensitivity analysis (GSA) using the R package pksensi [29]. We varied all parameters by 1% to compute the effect on NFT kinetics in plasma, and sensitivity coefficients over time were calculated for each parameter. We considered sensitivity coefficients higher than 0.1 as highly influential parameters and lower than 0.05 as non-influential parameters.

## 3. Results

### 3.1. Glomerular Filtration Is Insufficient to Describe Rabbit Plasma and Urine NFT Kinetics

In the literature, several data sets have been published over the past decades in which NFT was administered to rabbits [17], rats [23,28], and humans [4]. We digitized these data from the original publications, so they could be compared to PBPK model predictions. We started with PBPK model development on the basis of the extracted rabbit data, because for this species, both plasma and urine kinetic data following IV exposure to various doses of NFT were available. In order to explain these published rabbit data, we developed a PBPK model that included both liver metabolism and renal clearance via glomerular filtration (model V1). We calculated the tissue:plasma partition coefficients using the Berezhkovskiy method [24]. We then calibrated the remaining model parameters related to liver metabolism, the Restbody:plasma partition coefficient, and the urine elimination rate using the plasma and urine experimental data.

**Figure 2:**
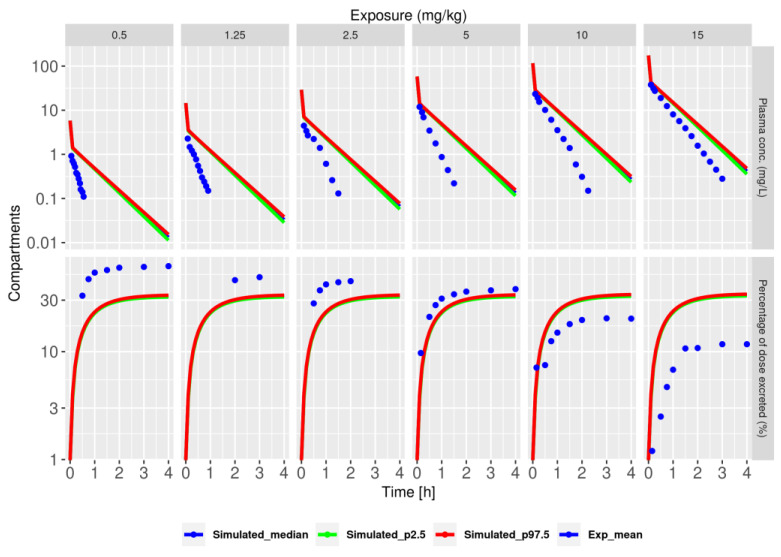
Model with only glomerular filtration (model V1) does not accurately describe NFT kinetics in plasma and urine. Plots show model simulations of the 2.5th (green), median (blue), and 97.5th (red) percentiles. The simulations were calculated based on 2000 randomly sampled parameter sets from the posterior distribution. Blue dots represent the mean of the experimental data. The top row corresponds to plasma, and the bottom row corresponds to urine, with exposure levels indicated above the panels in mg/kg.

The model failed to explain both the plasma and urine experimental data (Figure 2). Specifically, the plasma concentrations over time were overpredicted at all the doses. Importantly, the model also failed to predict the dose-dependent decrease in the percentage of elimination observed in the experimental data. For example, the model predicted that approximately 35% of the dose would be excreted regardless of the dose administered, while the experimental data showed a decrease from 60% to approximately 10%, with a 30-fold dose increase. In conclusion, the clearance mechanism does not linearly depend on the dose, and a simple model with glomerular filtration is insufficient to describe NFT kinetics.

### 3.2. Tubular Secretion and Reabsorption Jointly Explaining Dose-Dependent NFT Kinetics

Additional important processes in the kidney include active secretion and reabsorption, which can also be of relevance for administered drugs. To improve the description of the data, we created two modified models (model V2 and model V3). Model V2 included a saturated active secretion process, whereas model V3 instead added a first-order reabsorption process, in both cases on top of glomerular filtration. We hypothesized that increasing concentrations would lead to a saturation of active secretion and thus low relative excretion at high doses for model V2. For model V3, we expected a dose-dependent increase in reabsorption from the tubules back into the systemic circulation, resulting in a low amount of NFT available for excretion. Model V2 with active secretion resulted in better prediction, capturing the dose-dependent decrease in urine excretion compared to model V1. Nevertheless, at high doses, the model still underpredicted and overpredicted the plasma and urine kinetics, respectively (Figure S1A). Model V3 failed to improve the description of experimental data compared to model 1 (Figure S1B).

**Figure 3:**
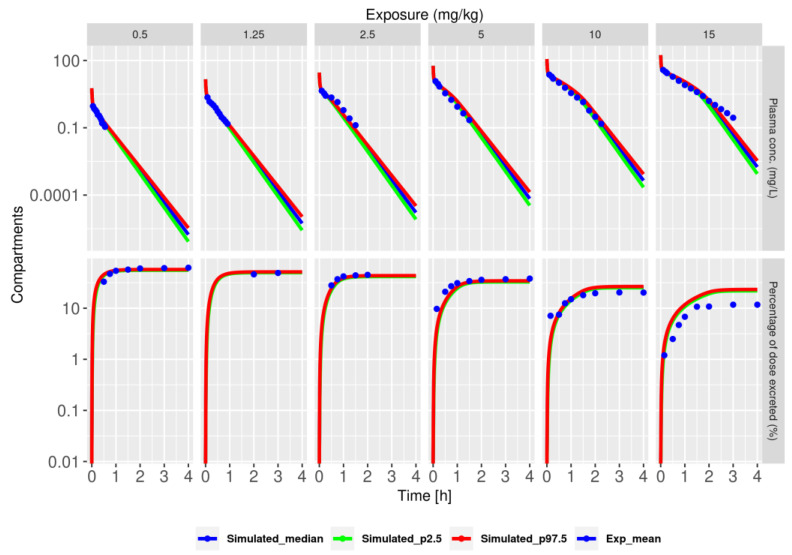
Model with active tubular secretion and reabsorption (model V4) describes plasma and urine NFT kinetics well. Plots show model simulations of the 2.5th (green), median (blue), and 97.5th (red) percentiles. The simulations were calculated based on 2000 randomly sampled parameter sets from the posterior distribution. Blue dots represent the mean of the experimental data. The top row corresponds to plasma, and the bottom row corresponds to urine, with exposure levels indicated above the panels in mg/kg.

We then hypothesized that both active tubular secretion and reabsorption could be important for renal clearance of NFT. Therefore, we extended the base model with both processes (referred to as model V4) and calibrated the parameters of both processes. The fitted model explained the dose-dependent urine and plasma kinetics quite well, outperforming the model with only active tubular secretion (Figure 3), with a slight underprediction of plasma concentrations and overprediction of urine kinetic data at the highest dose (maximally a 2-fold difference).

**Figure 4:**
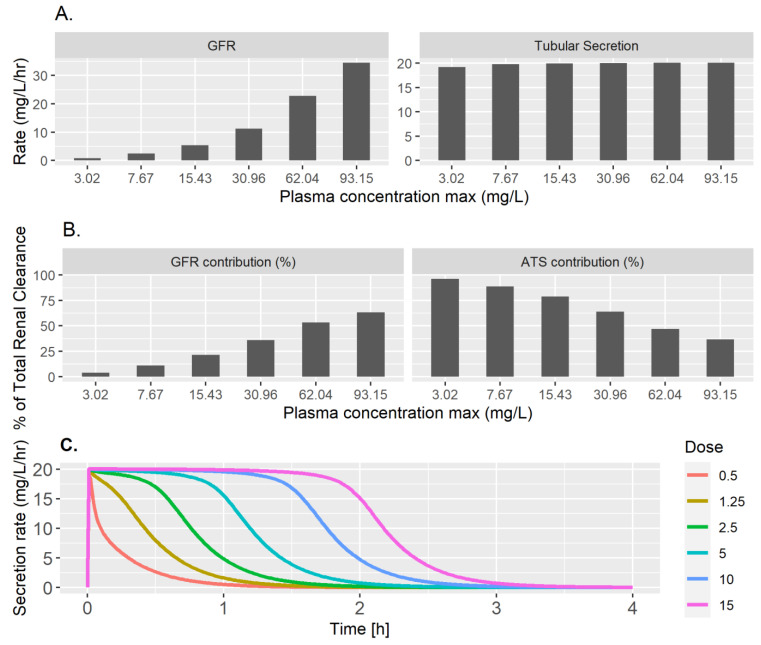
Concentration dependence of renal clearance after IV NFT dosing in rabbits. (**A**) The glomerular filtration rate (GFR, left) and the active tubular secretion rate (right) at the maximum plasma concentrations for six different IV doses: 0.5, 1.25, 2.5, 5, 10, and 15 mg/kg. (**B**) The percentage of total renal clearance contributed by the GFR and active tubular secretion (ATS) at the maximal plasma concentration for each of the six doses. In (**A**,**B**), the bars are arranged from left to right in increasing order of dose, with the first bar corresponding to the lowest dose of 0.5 mg/kg on the left and the sixth bar corresponding to the highest dose of 15 mg/kg on the right. (**C**) The active tubular secretion rate over time (x-axis) for six different IV doses (in mg/kg; colors indicated in the legend).

To further investigate the dose- and time-dependent relationship between the GFR and active secretion, we plotted the glomerular filtration rate and active tubular secretion rate against the maximal plasma concentration at each dose (Figure 4A). As expected, clearance via the GFR increased linearly with increasing concentration. In contrast, active secretion saturated at low concentrations, thus contributing to the dose dependency of NFT arrival in the urine. Consistently, the relative contribution of the GFR to the total renal clearance increased with the maximal plasma concentration and the relative contribution of active tubular secretion (ATS) decreased (Figure 4B). These results suggest that even at low plasma NFT concentrations, active secretion of NFT saturates already (i.e., the Km value is low), limiting NFT elimination from the body. The saturation of the active secretion rate with increasing dose was also visible from a time course plot of the tubular secretion rate and was especially clear at early time points (Figure 4C). In conclusion, saturation of active secretion at low NFT concentrations is necessary to explain the non-linear, dose-dependent kinetics in urine.

### 3.3. Renal Clearance and EHR Successfully Describe NFT Kinetics in Rats and Rabbits

We next asked whether the aforementioned PBPK model (model V4, fitted with rabbit data) could also be applied to rats following allometric scaling, for which only a limited number of time course measurements in plasma and urine were available from published data. The model was nicely able to explain the observed plasma concentrations and the dose-dependent decrease in the percentage excreted in urine at different IV doses (Figure S2). Specifically, both the model and the experimental data showed that at the lowest dose (3 mg/kg), around 40% was excreted, and this percentage decreased to around 25% at the highest dose of 25 mg/kg (Figure S2). The fact that the model was extrapolated from rabbits by adapting to known rat physiology and that biochemical parameters were scaled just based on rat body weight provides strong confidence in the developed PBPK model.

Nevertheless, the rat kinetics study from which we extracted the NFT data [23] showed that NFT is also excreted via bile. Therefore, we created another version of the model, where we included bile as a compartment, and we included EHR in the model, leading to model V5. We used both plasma and biliary excretion data upon IV dosing as inputs, and calibrated EHR-related parameters, while keeping the other parameters the same. The model extended with EHR could describe the biliary excretion data well and also resulted in a slight improvement in urine kinetics data (Figure S3). Interestingly, the simulated plasma kinetics data now exhibited biphasic kinetics due to recirculation between gut, bile, and liver. However, this cannot be validated in the absence of data at late time points.

**Figure 5:**
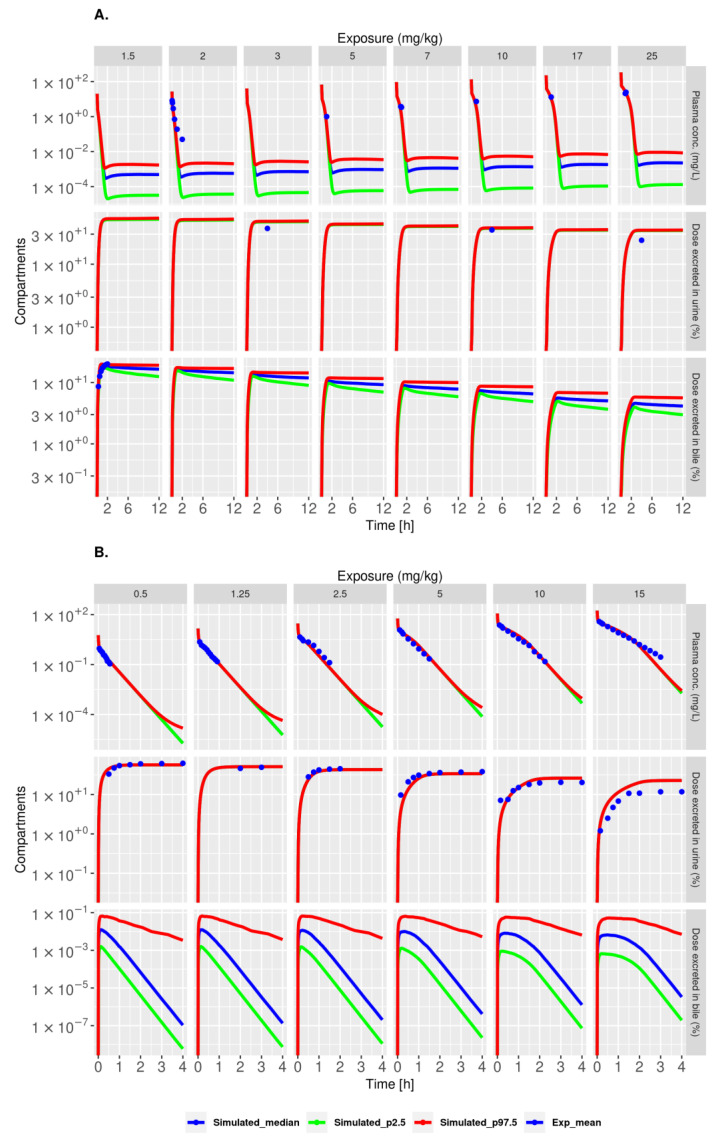
Model with both renal clearance and enterohepatic recirculation (model V5a) describes NFT kinetics in rats and rabbits after IV dosing. (**A**,**B**) Plots show model simulations of the 2.5th (green), median (blue), and 97.5th (red) percentiles for rats (**A**) and rabbits (**B**). The simulations were calculated based on 2000 randomly sampled parameter sets from the posterior distribution. Blue dots represent the mean of the experimental data. The top rows correspond to plasma, the middle rows to urine, and the bottom rows to bile, with exposure levels indicated above the panels in mg/kg.

With model V5 (extended with EHR), we then revisited the plasma and urine kinetics data upon IV dosing in rabbits. We incorporated the fitted EHR parameters based on rats into the simulations for rabbits, while keeping the other rabbit-specific parameters the same. The model nicely explained the plasma kinetics, but the percentage of dose excreted via urine especially was underpredicted at a low dose and overpredicted at a high dose (Figure S4). One factor determining this mismatch could be that due to the excretion of NFT into bile, the drug circulates systemically at lower concentrations than without EHR, leading to less urine excretion at low doses. Moreover, the limited data availability with respect to biliary excretion in rats (only for one dose) and the absence of such data in rabbits might have led to poorly predicted urine data. Finally, biliary excretion may be species specific, e.g., due to the distinct expression of specific transporters [30]. To overcome these limitations, we applied hierarchical modeling to the EHR-related parameters on the basis of both rat and rabbit experimental data (including both IV and oral dosing data). This improved the prediction of the IV dosing data for both species (Figure 5). The model predicted a dose-dependent relative decrease in biliary excretion in both rats and rabbits, with low relative biliary excretion in rabbits. Moreover, the model prediction of plasma concentrations over time also reasonably agreed with the experimental data following oral dosing, although the model predicted slightly too fast uptake and underestimated the late time points for rat kinetics (Figures S5 and S6). In addition, we performed an in silico knockout of the EHR process (no efflux from the liver to bile) in both rabbits and rats (Figure S7). This resulted in linear plasma elimination kinetics (losing the property of biphasic kinetics), with little or no effect on urine kinetics. A global sensitivity analysis of rabbit model 5a upon IV dosing of 15 mg/kg showed all parameters to be sensitive for all the compartments (Figure S8). In conclusion, by including EHR into our PBPK model on the basis of rat measurements, we obtained acceptable descriptions of both rat and rabbit NFT kinetics data for both IV and oral dosing, suggesting that the most important processes for NFT absorption, distribution, metabolism, and excretion (ADME) are covered by the PBPK model.

### 3.4. Extrapolation of PBPK Model from Rats and Rabbits to Humans

Having a PBPK model for NFT that integrated both renal clearance and EHR, we next extrapolated this model to humans. We implemented this by using human-specific physiology and by allometric scaling of renal clearance parameters, as estimated from the rabbit data, and of EHR and gut absorption parameters, as estimated from the rat data. To account for parameter variability, we ran 2000 simulations using individual parameter values drawn randomly around their fitted mean value, with a standard deviation of 1.17 (on a log scale), equivalent to a coefficient of variation (CoV) of 16%. This choice of CoV was informed by the estimated range of the posterior parameters and offers a reasonable range of variability around the mean. Note that detailed NFT measurements amongst multiple individuals would allow for a more data-informed choice of the CoV across individuals. We also varied age from 25 to 40 using a uniform distribution and scaled the human physiological parameters based on age (see Section 2).

**Figure 6:**
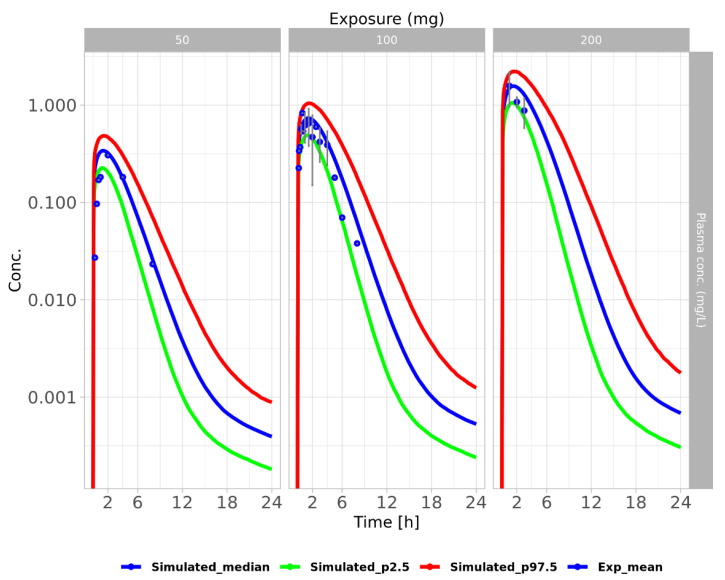
Cross-species extrapolated human PBPK model that describes nitrofurantoin concentration after a single oral dose. Plots display simulated NFT concentrations with 95% confidence intervals of the 2.5th (green), median (blue), and 97.5th (red) percentiles. The simulated data are based on 2000 iterations using individual parameter values drawn randomly around their fitted mean value, with a standard deviation of 1.17. Experimental mean data are represented by blue dots in plasma at different doses, indicated above the panels in mg.

Based on the extrapolated model (i.e., without further model calibration) and using oral doses as input, we predicted the kinetics of NFT in human plasma and compared these to published measurements from [4] (Figure 6). The model captured the dose-dependent increase in the plasma concentration well. The close agreement between experimental data and model prediction provided strong confidence in the PBPK model. We observed only a minor dose-dependent decrease in the percentage of the dose excreted into urine in human PBPK simulations (Figure S9). Moreover, for the applied dose range, only a low amount of NFT was excreted into bile (between 0.2 and 1.5%) (Figure S9). NFT was thus predicted to be quickly eliminated from all organs (within 12 h), and NFT maximal concentrations in the various organs followed this order: liver > kidney > tubules > plasma > rest of the body > fat (Figure S10).

### 3.5. Influence of GFR on Excretion and Build-Up of Systemic NFT Concentration

NFT is used as an antimicrobial agent for UTIs; hence, after oral administration, it is aimed to act against microbial infections in the bladder. The arrival of NFT at that anticipated location could be influenced by the exact physiological parameters of individuals. One important factor could be the GFR, which is known to decrease with age [31,32], and this could lead to potentially toxic concentrations within organs, such as the liver. Therefore, we used our extrapolated human PBPK model to investigate the effect of the GFR on NFT concentrations in various organs.

**Figure 7:**
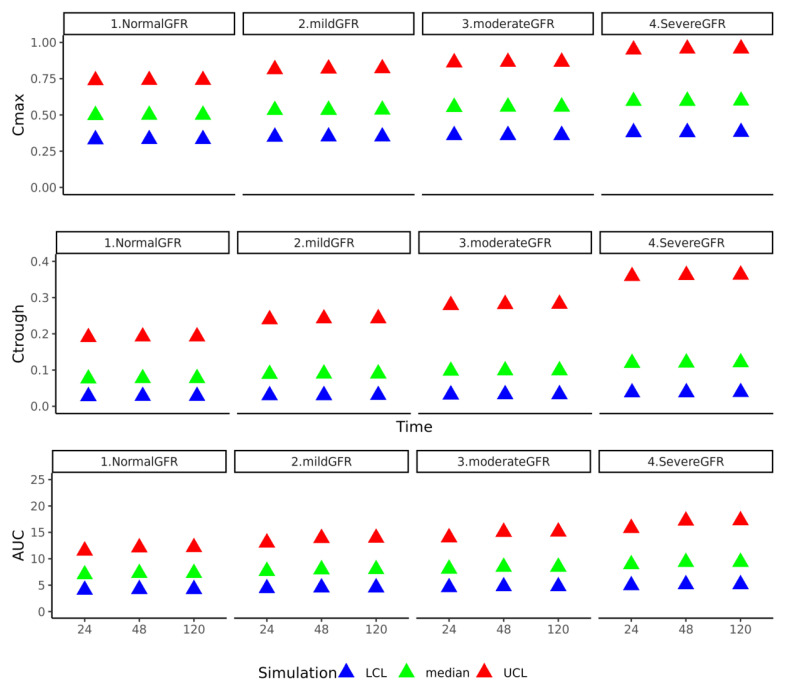
Predicted influence of GFR condition on NFT plasma concentrations. The human PBPK model V5a was simulated with oral NFT dosing (50 mg) four times a day. Each row represents one PK parameter (Cmax, Ctrough, and AUC) determined from time course simulations and is plotted at three different time points (24 h, 48 h, and 120 h). GFR conditions used (indicated above panels): normal GFR (>90 mL/min), moderate GFR (70 mL/min), mild GFR (45 mL/min), and severe GFR (20 mL/min). The shapes correspond to minimal (blue triangle), maximal (red triangle), and mean (green triangle) calculated based on 2000 simulations using individual parameter values drawn randomly from a Gaussian distribution centered around the fitted mean value per parameter (with a standard deviation of 1.17 on a log scale). Note that the predicted impact of GFR conditions on NFT concentrations in the liver and urine are shown in Figures S11 and S12.

We considered a typical oral dosage regimen of 50 mg administered four times a day for five days. We calculated the maximal concentration (Cmax), minimal concentration (Ctrough), and AUC for each day using the simulated NFT concentrations in the plasma over time and plotted them at three time points (day 1, day 2, and day 5) (Figure 7). A decrease in the GFR (from normal to severe GFR compromised) resulted in an approximately 1.3-fold increase in Cmax and a 2-fold increase in Ctrough, i.e., the entire Cmax–Ctrough window shifted to a higher level. Moreover, effective exposure to the plasma also increased, as shown by approx. a 1.3-fold difference in the AUC when comparing normal and severe GFR conditions. We observed similar results for the liver (Figure S11). As expected, the compromised GFR conditions resulted in substantial reductions in the percentage of dose excreted in urine (Figure S12). In conclusion, our PBPK model predicts that in people with a compromised GFR, treatment with NFT might not be effective due to the low arrival of NFT in the bladder. Moreover, in such individuals, NFT concentrations in the liver rise to levels that may be toxic.

## 4. Discussion

In this study we developed a PBPK model for NFT kinetics using published data from rabbits and rats and extrapolated the model to predict human kinetics. NFT is a commonly used oral antibiotic for treating low UTIs, but it is associated with the occurrence of drug-induced liver injury (DILI), which is the most common adverse effect of NFT exposure. The mechanisms leading to DILI are not completely clear, and severe fatalities due to hepatic toxicity have been reported for NFT. Animal PK studies have indicated that NFT kinetics are not linearly proportional to the administered dose [12,17,19]. At the same time, human PK exhibits high variability [2,4,33,34]. Thus, there is a strong need to understand the relationship between physiological factors, NFT exposure, and its disposition, as this might help us to understand the quantitative contribution of kinetics to the risk of hepatic adverse events.

We created a series of PBPK models of increasing complexity to capture NFT kinetics in the body as realistically as possible on the basis of available data. Our initial model included only liver metabolism and renal clearance via glomerular filtration. To improve the prediction of dose-dependent effects, we created various modified models, including active tubular secretion and/or reabsorption. A PBPK model with both these processes included described the plasma and urine kinetics data much better than models with none or only one of these processes included. Mechanistic analysis of the resulting model suggested that active secretion of NFT saturates already at low plasma concentrations, limiting NFT elimination from the body primarily via such active secretion. Together with a high rate of reabsorption, this saturated active secretion at low concentrations was necessary to explain the non-linear, dose-dependent kinetics in urine. These findings are in line with classical dog studies in which NFT tubular secretion saturated and reabsorption occurred at a high rate [19,35].

Excretion of NFT is not only mediated by the kidney but also by hepatobiliary elimination. In mice and rats, the ABC transporter BCRP1 is involved in the latter elimination route [20]. Oo et al. (2001) showed that BCRP1 plays a role in humans as well (specifically for secretion of NFT into human milk), suggesting that this transporter promotes hepatobiliary excretion in humans [36]. Therefore, we extended our PBPK model with bile as an additional bile compartment, which could also describe kinetics data in all species. Calibration of our PBPK model with EHR predicted a dose-dependent relative decrease in biliary excretion in both rats and rabbits; however, in rabbits this excretion appears to be low (less than 1%), suggesting that EHR is species specific. However, further data are needed to support this result.

Our extrapolated human PBPK model (model V5a) nicely captured the dose-dependent increase in human plasma kinetics data for three different doses. The model predicted NFT to be quickly eliminated from all organs (Figure S10), which is in line with the NFT half-life [35]. However, the model predicted less than 2% of administered NFT was excreted into bile, indicating that this excretion route is not so relevant in humans (as in rabbits). Consistently, C421A polymorphism in ABCG2 (gene name for BCRP1) had no effect on NFT plasma and urine PK parameters, at least in healthy subjects [37]. Further, in silico knockout of EHR showed no effect on urine excretion (Figure S7), and this is in line with experiments with BCRP1-knockout mice, showing that such knockout does not affect urine NFT excretion [12]. However, the role of BCRP1 in both intestinal absorption and biliary excretion needs further study because BCRP1 expression is population and age dependent [38].

To arrive at the prediction of NFT kinetics in individual humans, variability in enzymes involved in drug metabolism, such as CYP enzymes, transporters (like the mentioned BCRP1), and physiological parameters need to be taken into account, factors that are typically also age dependent. Such an approach could provide individualized dosing advice and predict effectivity and potential adverse outcomes. Here, we took a simpler approach by studying various scenarios of patients with compromised GFRs based on our human PBPK model, i.e., by varying this important age-dependent parameter across patient subgroups. This analysis indicated that individuals with a moderate-to-severe GFR are expected to be exposed to a 1.3-to-2-fold higher concentration of NFT than people with a normal GFR. Moreover, our model predicted a substantial reduction in NFT excreted into urine (approximately 30% less) in GFR-compromised individuals. Collectively, these results have important clinical implications, because individuals with renal insufficiency may be at a high risk of developing DILI due to increased exposure to NFT. At the same time, NFT treatment of UTIs in GFR-compromised individuals may well result in a suboptimal therapeutic effect and is thus associated with a high risk for drug resistance. This finding is in line with the contraindication for usage of NFT in individual with a GFR of less than 60 mL/min [14,16].

One limitation of this study is the lack of information in humans regarding the role of transporters in NFT renal and biliary clearance. Furthermore, we simplified the oral absorption process by not taking into account the gastric emptying time, and drug formulation characteristics, such as tablets with sustained release, that could result in PK variability [34]. In addition, studies in mice and rats have shown that BCRP could also play a role in intestinal absorption, which might result in non-linear PK absorption [22,23]. This could be one reason why the model did predict rat and rabbit oral kinetics well. Finally, in vitro data on liver metabolism, intestinal absorption, and renal elimination were not available. Instead, we determined the values for a subset of the parameters with top-down-approach PBPK (i.e., by parameter calibration), which led to good fitting and mechanistic insight. Nevertheless, future in vitro data on the mentioned processes would likely further improve our model and ameliorate parameter uncertainty.

In conclusion, our developed PBPK model for NFT provides a valuable tool to predict the consequences of NFT exposure and demonstrates the utility of PBPK modeling in understanding NFT pharmacokinetics, especially in patients with renal impairments. Our work highlights the probable importance of active tubular secretion and reabsorption in explaining the dose-dependent effects of NFT, and they have important clinical implications for the safe and effective use of the drug. This improves our understanding of the relationship between NFT exposure, its disposition, and the occurrence of adverse events, such as DILI and lung toxicity. Note that to arrive at predictions for lung toxicity, the PBPK model should first be extended with a lung compartment. Future studies could furthermore focus on refining the model further by incorporating additional data on biliary excretion and other factors that could impact NFT disposition, particularly in specific populations. The model can also be further coupled with computational models describing various downstream effects, such as NFT-induced stress pathway activity within the liver. Such stress pathway activity is known to vary strongly amongst individuals [39,40], which may contribute to liver toxicity in specific subpopulations independent of renal impairment. In the case of NFT exposure, this concerns especially NRF2- and ATF4-mediated signaling [11]; hence, the expression of proteins involved in these pathways might represent additional risk factors. Future integration of our PBPK model with stress pathway models may allow the prediction of the effect of interindividual variability, renal function, and various dosage regimens on NFT disposition, stress biomarkers, and potential liver injury.

## Data Availability

No new data were created or analyzed in this study. Code to run the developed PBPK models is available at https://doi.org/10.5281/zenodo.8276305 (released on 23 August 2023).

## Supplementary Materials

The following supporting information can be downloaded at: https://www.mdpi.com/article/10.3390/pharmaceutics15092199/s1, Figure S1: Model with either active tubular secretion (Model V2) or tubular reabsorption (Model V3) does not accurately describe NFT kinetics in plasma and urine; Figure S2: Measured and simulated NFT concentrations in rats after IV dosing; Figure S3: Measurements and simulations of NFT concentrations in rats after IV dosing using Model V5; Figure S4: Measurements and simulations of NFT concentrations in rabbits after IV dosing using Model V5; Figure S5: Measurements and simulations of NFT concentrations in rabbits after oral dosing using Model V5a; Figure S6: Measurements and simulations of NFT concentrations in rats after oral dosing using Model V5a; Figure S7: In-silico knock out of EHR process in (Model V5a) in rats and rabbits after IV dosing; Figure S8: Sensitivity of PBPK model parameters; Figure S9: Simulations of excreted NFT in humans after oral dosing using extrapolated ModelV5a; Figure S10: Simulations of NFT concentrations in various human tissues after oral dosing using extrapolated ModelV5a; Figure S11: Predicted influence of GFR condition on NFT liver concentrations; Figure S12: Predicted influence of GFR condition on NFT plasma, liver, and urine concentrations; Table S1: Rat physiological parameters; Table S2: Rabbit physiological parameters; Table S3: Human adult physiological parameters; Table S4: PBPK biochemical parameters for three different species. References [17,41,42,43,44,45,46] are cited in the supplementary materials.
