# Supplementary material for: Development of a Physiologically Based Pharmacokinetic Model for Nitrofurantoin in Rabbits, Rats, and Humans
Source: Pharmaceutics. 2023 Aug 25;15(9):2199. doi: 10.3390/pharmaceutics15092199 (PMC10535763; doi:10.3390/pharmaceutics15092199)
Supplement: Supplementary file 1 [file pharmaceutics-15-02199-s001.zip › pharmaceutics-2496544-supplementary.pdf]

Article

Development of a Physiologically Based Pharmacokinetic Model for Nitrofurantoin in Rabbits, Rats, and Humans

Raju Prasad Sharma, Elsje J. Burgers and Joost B. Beltman *

Division of Drug Discovery and Safety, Leiden Academic Centre for Drug Research, Leiden University,
Einsteinweg 55, 2333 CC Leiden, The Netherlands; rpsharmasysbio@gmail.com (R.P.S.); e.j.burgers@lacdr.leidenuniv.nl (E.J.B.)
* Correspondence: j.b.beltman@lacdr.leidenuniv.nl

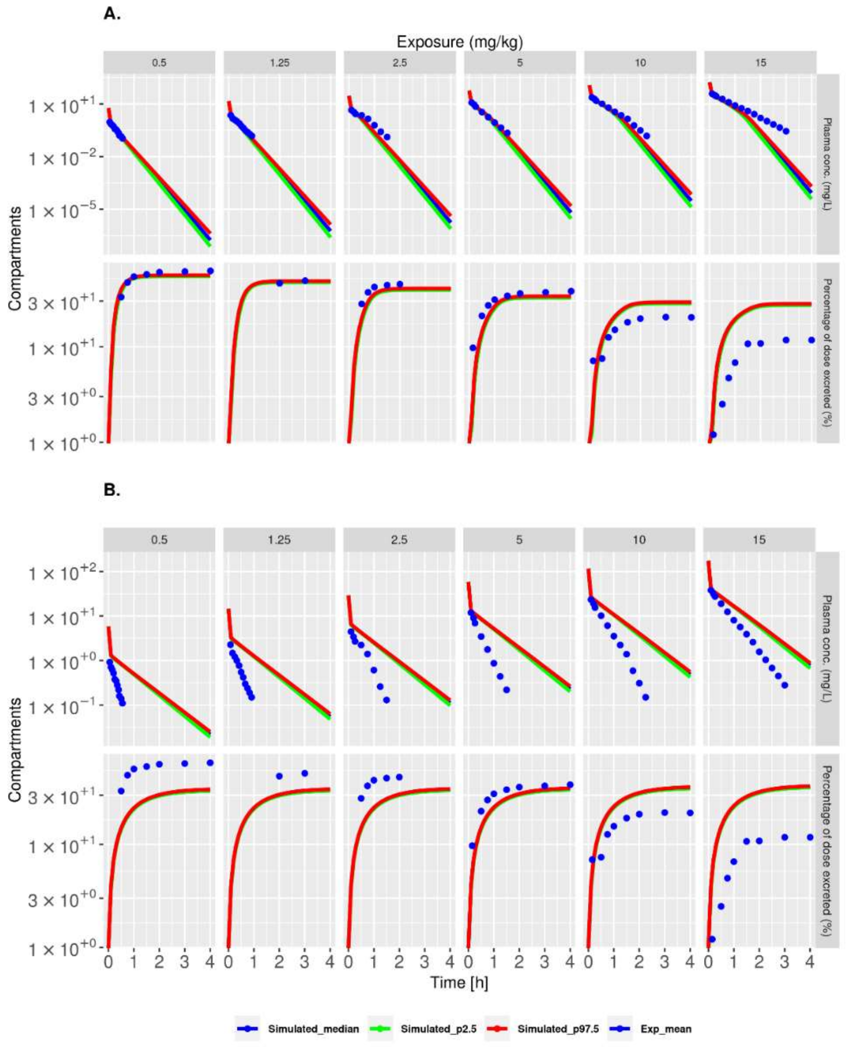

Figure S1. Model with either active tubular secretion (Model V2) or tubular reabsorption (Model V3) does not accurately describe NFT kinetics in plasma and urine. (A,B) Plots show model simulations of the 2.5th

(green), median (blue) and 97.5th (red) percentile for Model V2 with active tubular secretion (A) or with tubular reabsorption (B). The simulations were calculated based on 2000 randomly sampled parameter sets from the posterior distribution. Blue dots represent the mean of the experimental data. The top row corresponds to plasma and the bottom row corresponds to urine, with exposure levels indicated above the panels in mg/kg.

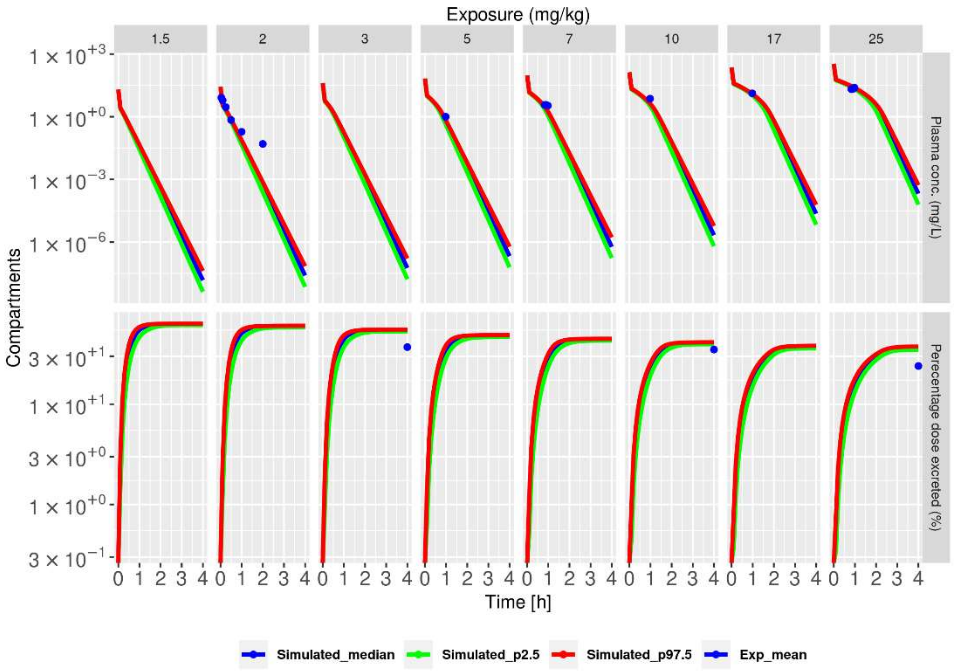

Figure S2. Measured and simulated NFT concentrations in rats after IV dosing. Simulations are based on extrapolation of Model V4 from rabbits to rats. Solid lines represent simulated 2.5th (green), median (blue) and 97.5th (red) percentile. The simulations were calculated based on 2000 randomly sampled parameter sets from the posterior distribution. Blue dots represent the mean of the experimental data. The top row corresponds to plasma and the bottom row corresponds to urine, with exposure levels indicated above the panels in mg/kg.

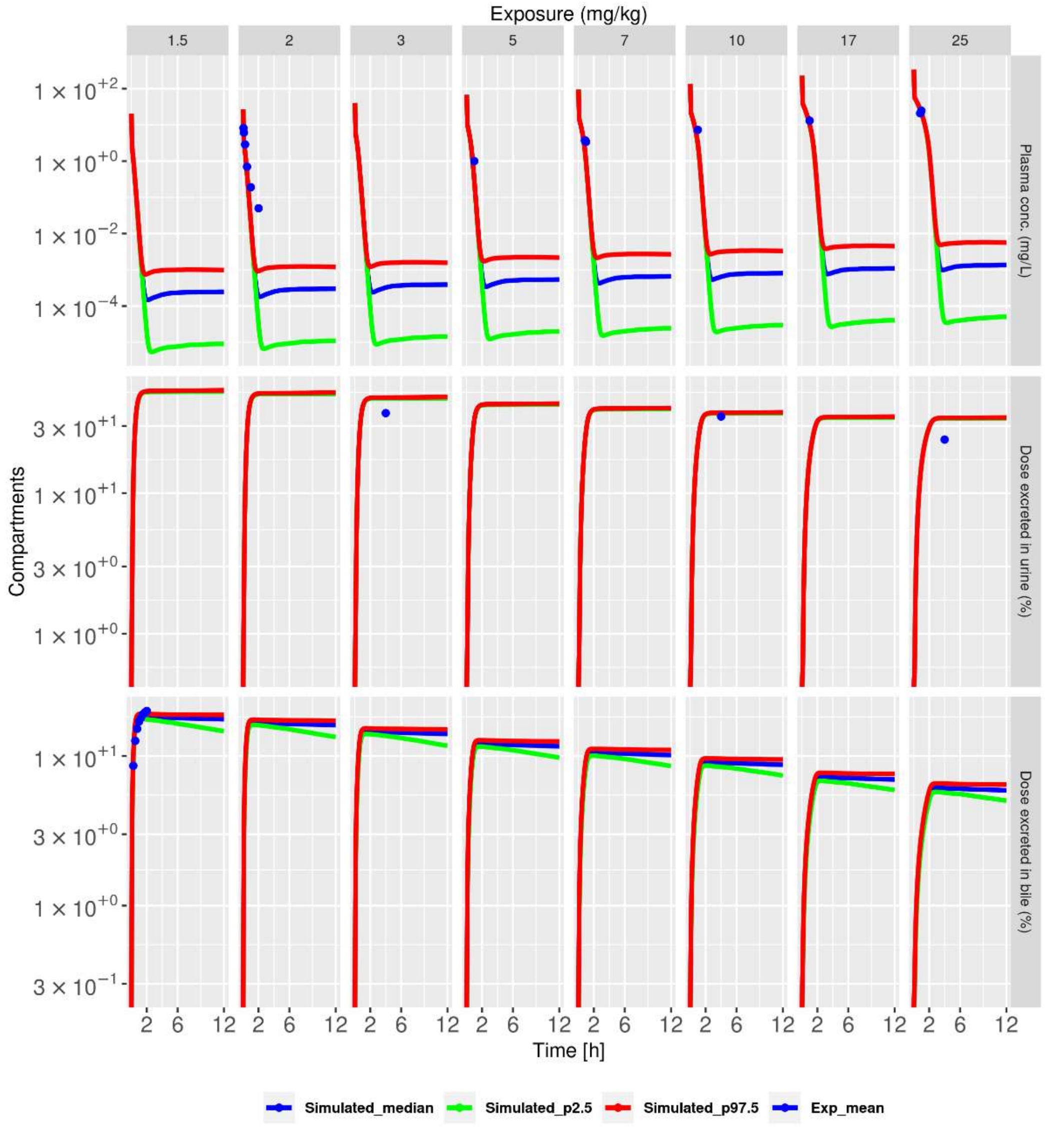

Figure S3. Measurements and simulations of NFT concentrations in rats after IV dosing using Model V5. Solid lines represent simulated 2.5th (green), median (blue) and 97.5th (red) percentile. The simulations were calculated based on 2000 randomly sampled parameter sets from the posterior distribution. Blue dots represent the mean of the experimental data. The top row corresponds to plasma and the bottom row corresponds to urine, with exposure levels indicated above the panels in mg/kg.

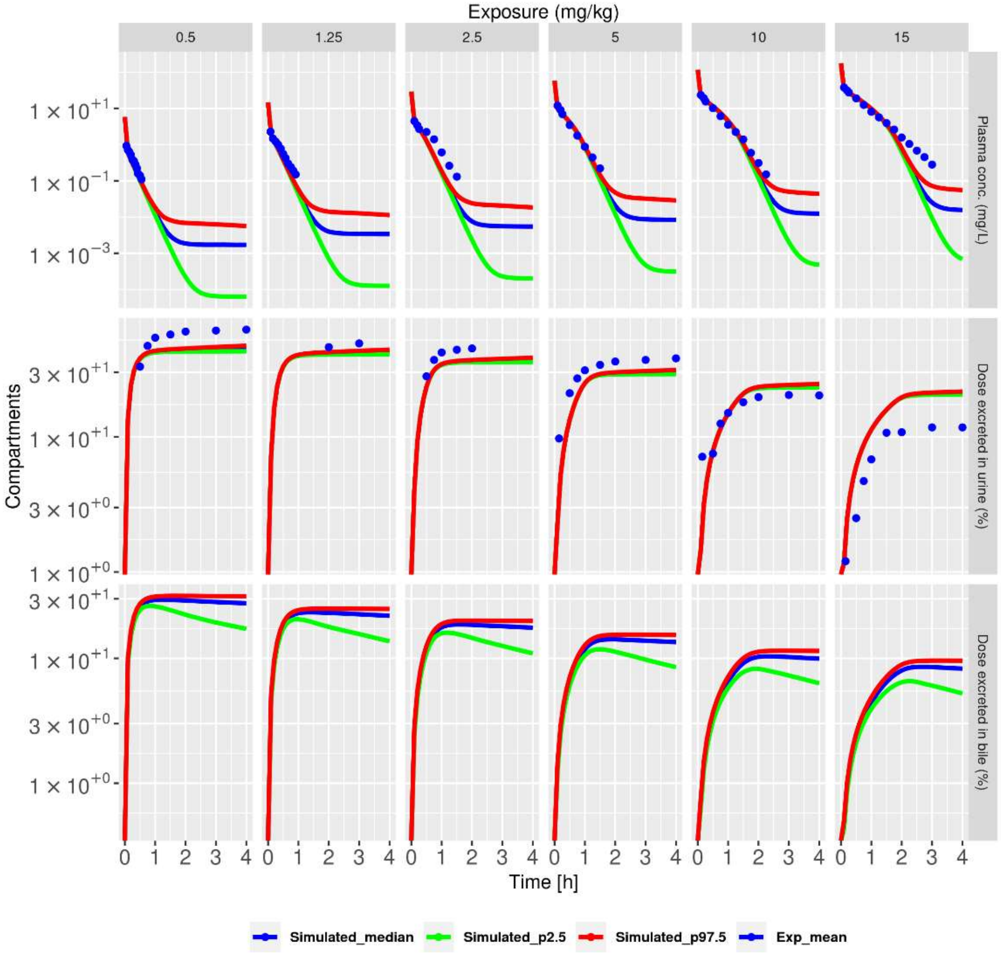

Figure S4. Measurements and simulations of NFT concentrations in rabbits after IV dosing using Model V5. Solid lines represent simulated 2.5th (green), median (blue) and 97.5th (red) percentile. The simulations were calculated based on 2000 randomly sampled parameter sets from the posterior distribution. Blue dots represent the mean of the experimental data. The top row corresponds to plasma, the middle row to urine, and the bottom row to bile, with exposure levels indicated above the panels in mg/kg.

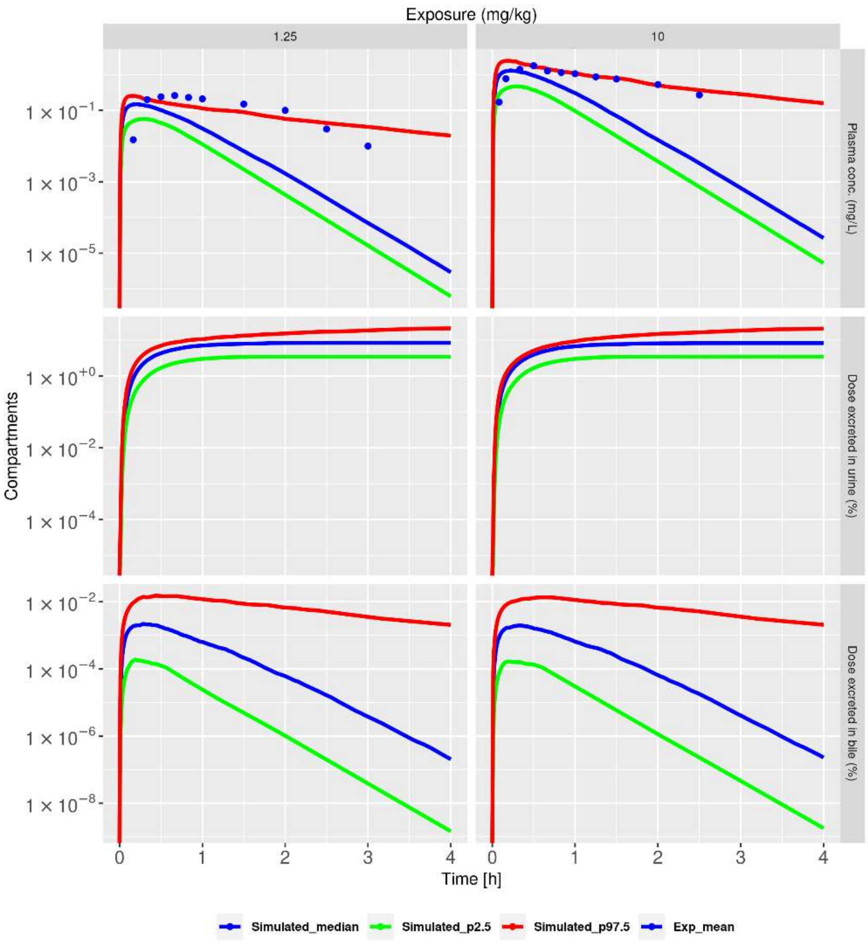

Figure S5. Measurements and simulations of NFT concentrations in rabbits after oral dosing using Model V5a. Solid lines represent simulated 2.5th (green), median (blue) and 97.5th (red) percentile. The simulations were calculated based on 2000 randomly sampled parameter sets from the posterior distribution. Blue dots represent the mean of the experimental data. The top row corresponds to plasma, the middle row to urine, and the bottom row to bile, with exposure levels indicated above the panels in mg/kg.

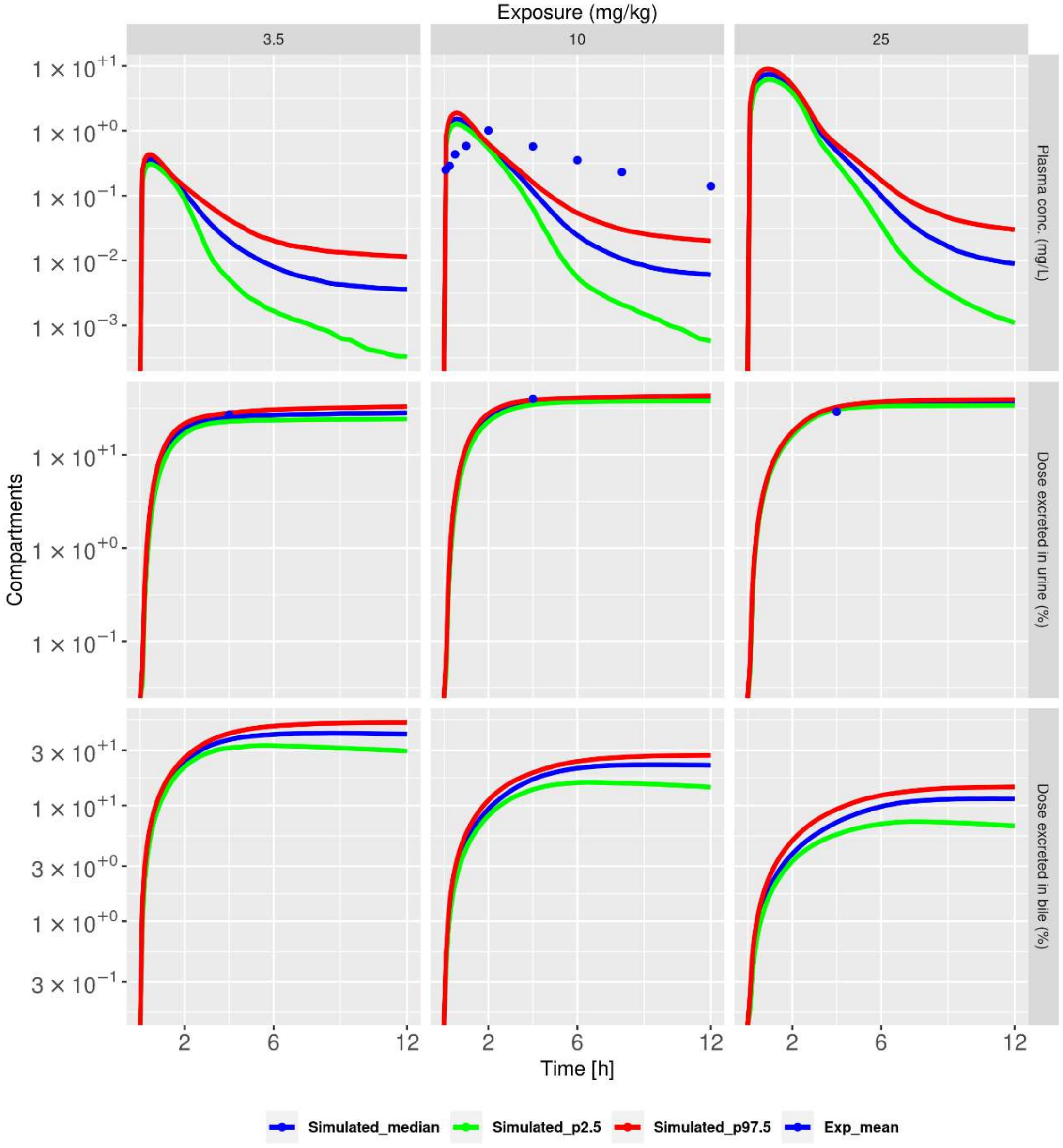

Figure S6. Measurements and simulations of NFT concentrations in rats after oral dosing using Model V5a. Solid lines represent 2.5th (green), median (blue) and 97.5th (red) percentile. The simulations were calculated based on 2000 randomly sampled parameter sets from the posterior distribution. Blue dots represent the mean of the experimental data. The top row corresponds to plasma, the middle row to urine, and the bottom row to bile, with exposure levels indicated above the panels in mg/kg.

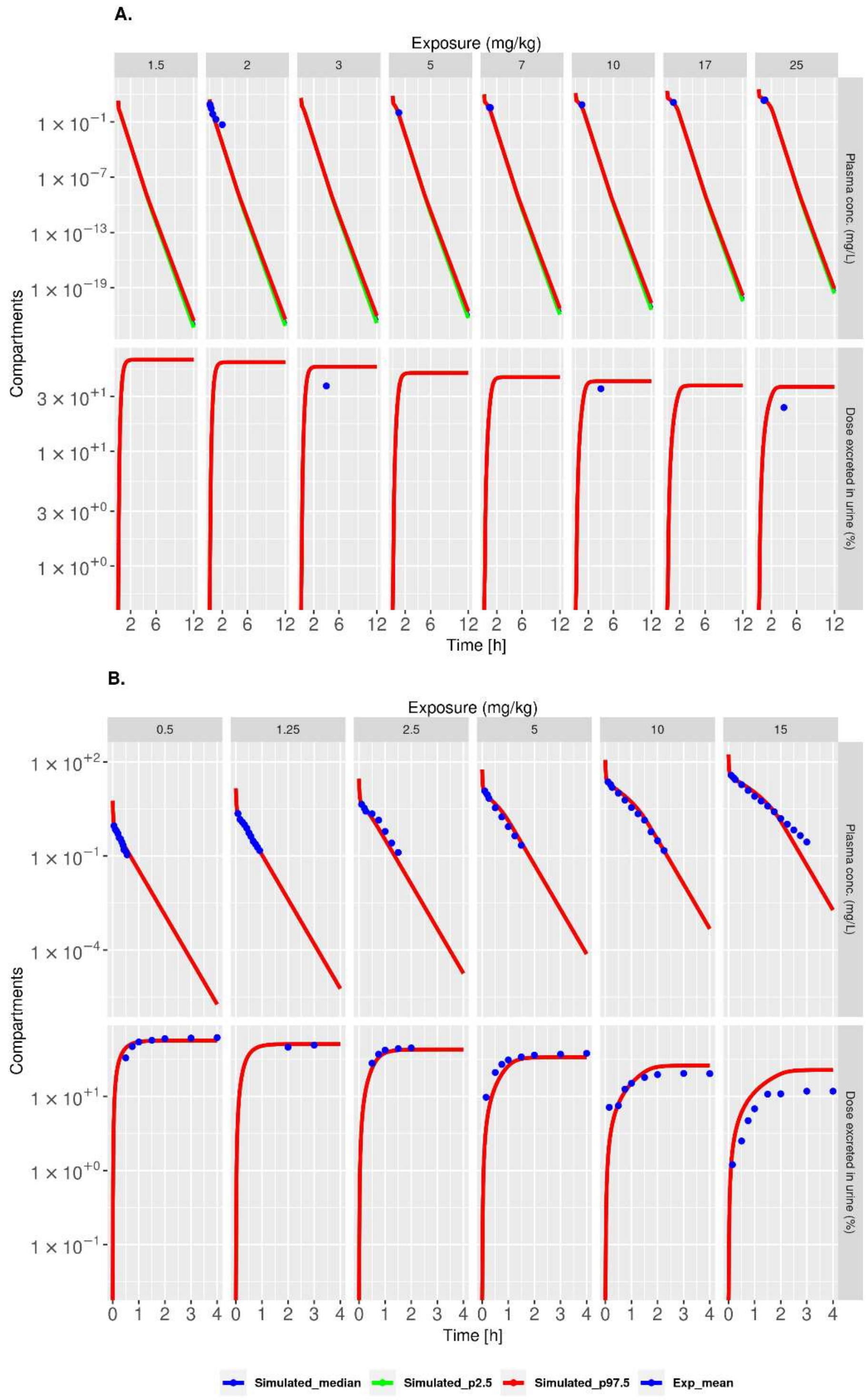

Figure S7. In-silico knock out of EHR process in (Model V5a) in rats and rabbits after IV dosing. (A,B) Plots show model simulations of the 2.5th (green), median (blue) and 97.5th (red) percentile for rats (A) and rabbits (B). The simulations were calculated based on 2000 randomly sampled parameter sets from the posterior distribution. Blue dots represent the mean of the experimental data. The top rows correspond to plasma and the bottom rows to urine, with exposure levels indicated above the panels in mg/kg.

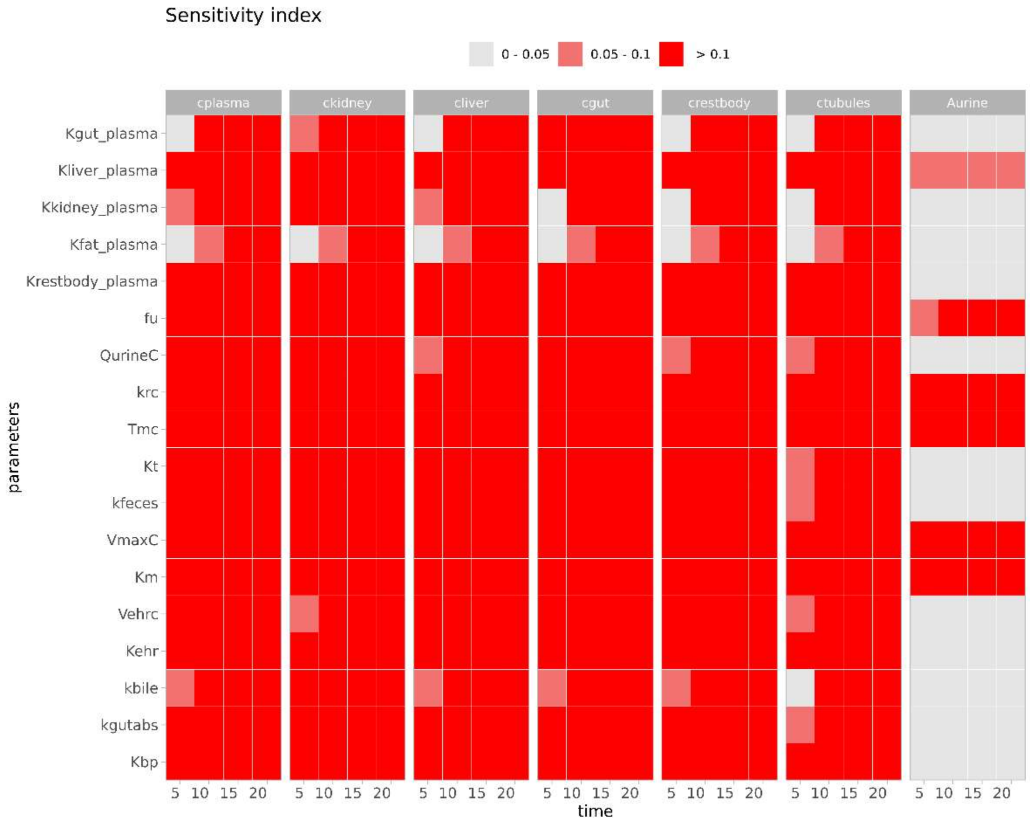

Figure S8. Sensitivity of PBPK model parameters. Plot shows a heatmap of time-dependent normalized sensitivity indices for all parameters on NFT PBPK output upon IV dosing of 15mg/kg in rabbits using Model V5a. Rows associate with model parameters and columns with model compartments (organs). Per column the horizontal axis describes the time in hours. The sensitivity index is categorized into three classes, as indicated by colour in the legend: (1) not sensitive when less than 0.05 (grey bars), (2) sensitive when value between 0.05-0.1 (light red bars), and (3) highly sensitive when greater than than 0.1 (dark red bars).

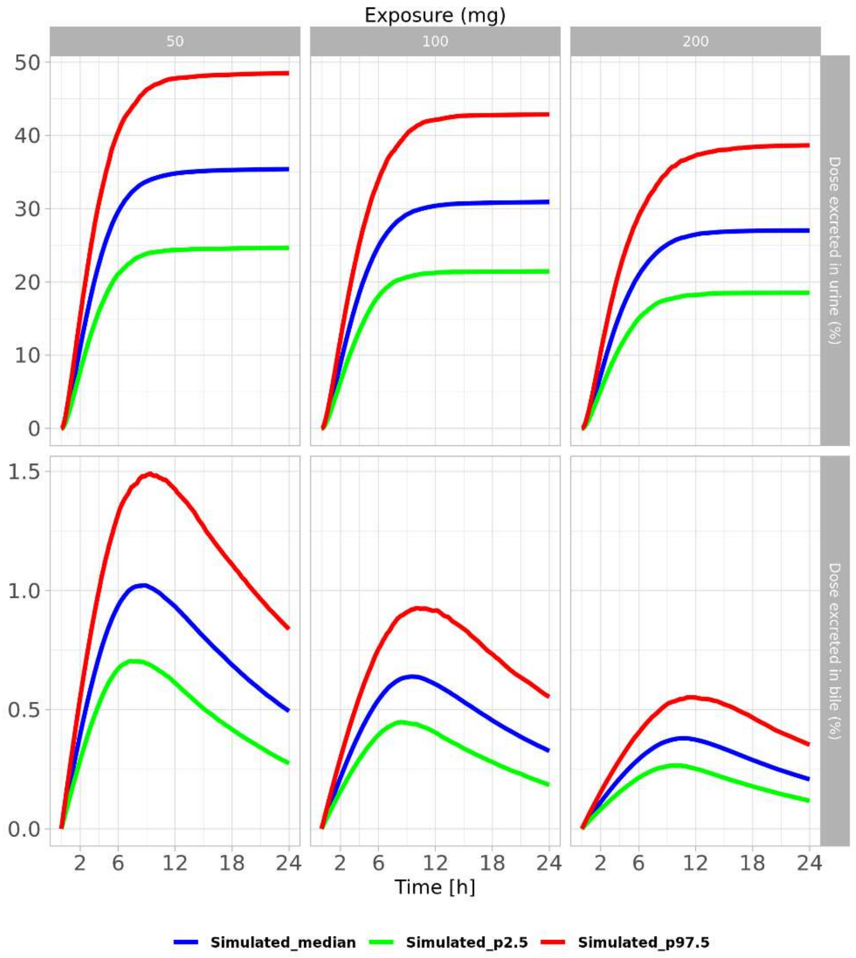

Figure S9. Simulations of excreted NFT in humans after oral dosing using extrapolated Model V5a. Solid lines represent simulated 2.5th (green), median (blue) and 97.5th (red) percentile. The simulations were calculated based on 2000 randomly sampled parameter sets from the posterior distribution. Blue dots represent the mean of the experimental data. The top row corresponds to urine, and the bottom row to bile, with exposure levels indicated above the panels in mg/kg.

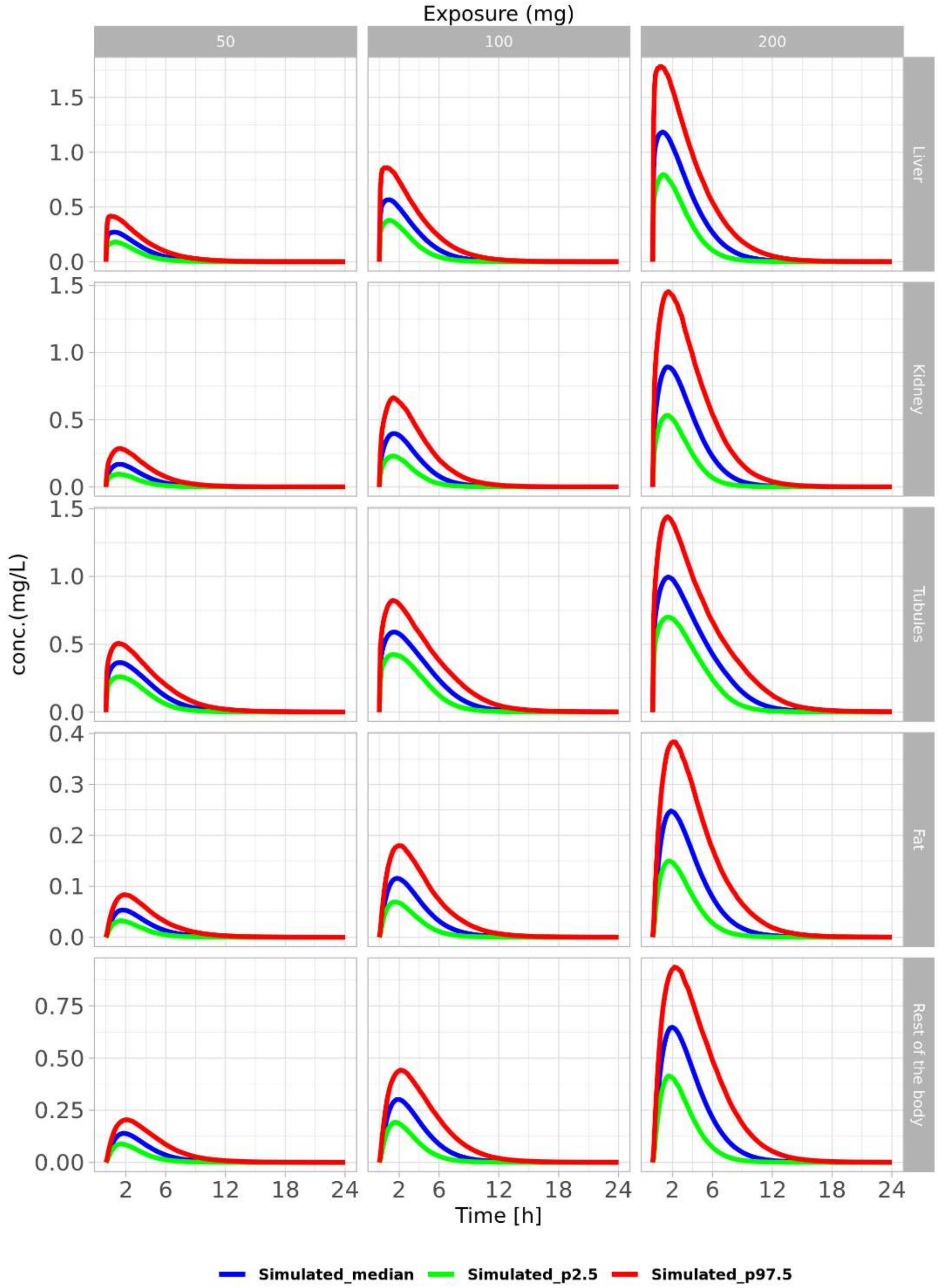

Figure S10. Simulations of NFT concentrations in various human tissues after oral dosing using extrapolated Model V5a. Solid lines represent simulated 2.5th (green), median (blue) and 97.5th (red) percentile. The simulations were calculated based on 2000 randomly sampled parameter sets from the posterior distribution. The simulated organs are indicated at the right panel of each plot, with exposure levels indicated above the panels in mg/kg.

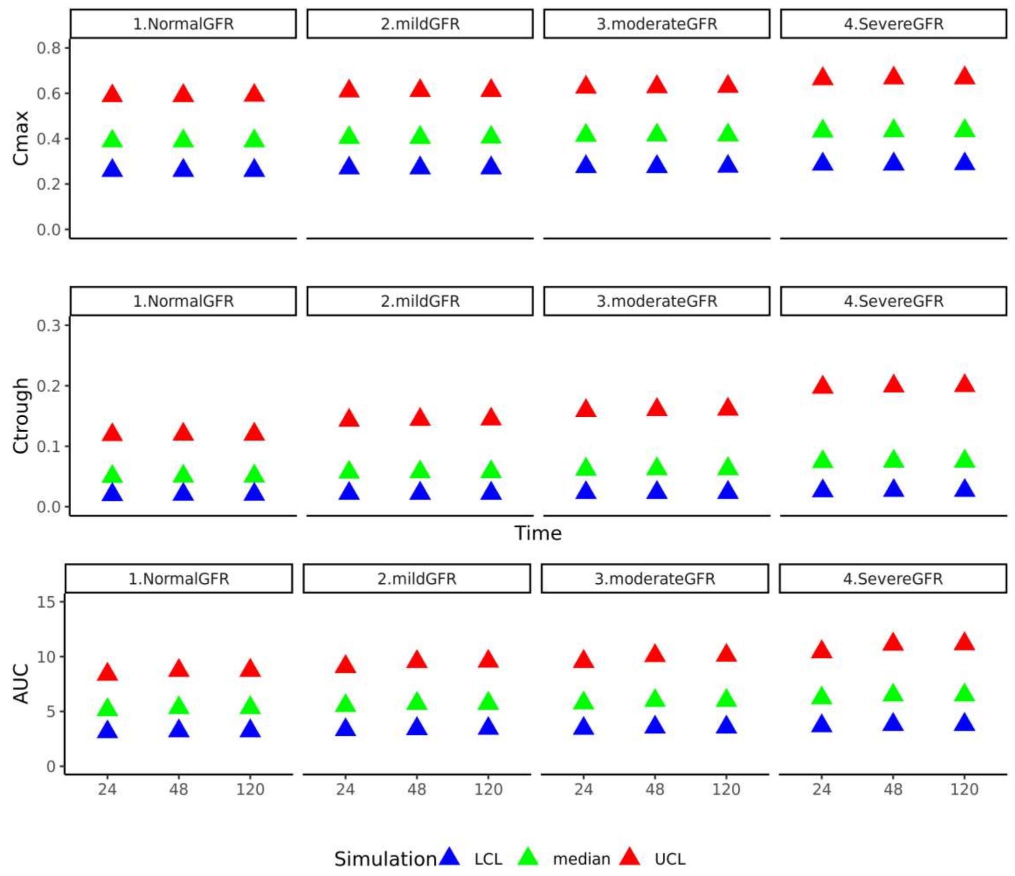

Figure S11. Predicted influence of GFR condition on NFT liver concentrations. The human PBPK model V5a was simulated with oral NFT dosing (50mg) four times a day. Each row represents one PK parameter (Cmax, Ctrough and AUC) determined from time course simulations, and is plotted at three different time points (24h, 48h, and 120h). GFR conditions utilized (indicated above panels): normal GFR (>90 mL/min), moderate GFR (70 ml/min), mild GFR (45 mL/min), severe GFR (20 mL/min). The shapes correspond to minimal (blue triangle), maximal (red triangle inside square) and mean (green circle) calculated based on 2000 simulations using individual parameter values drawn randomly from a Gaussian distribution centered around the fitted mean value per parameter (with a standard deviation of 1.17 on a log scale).

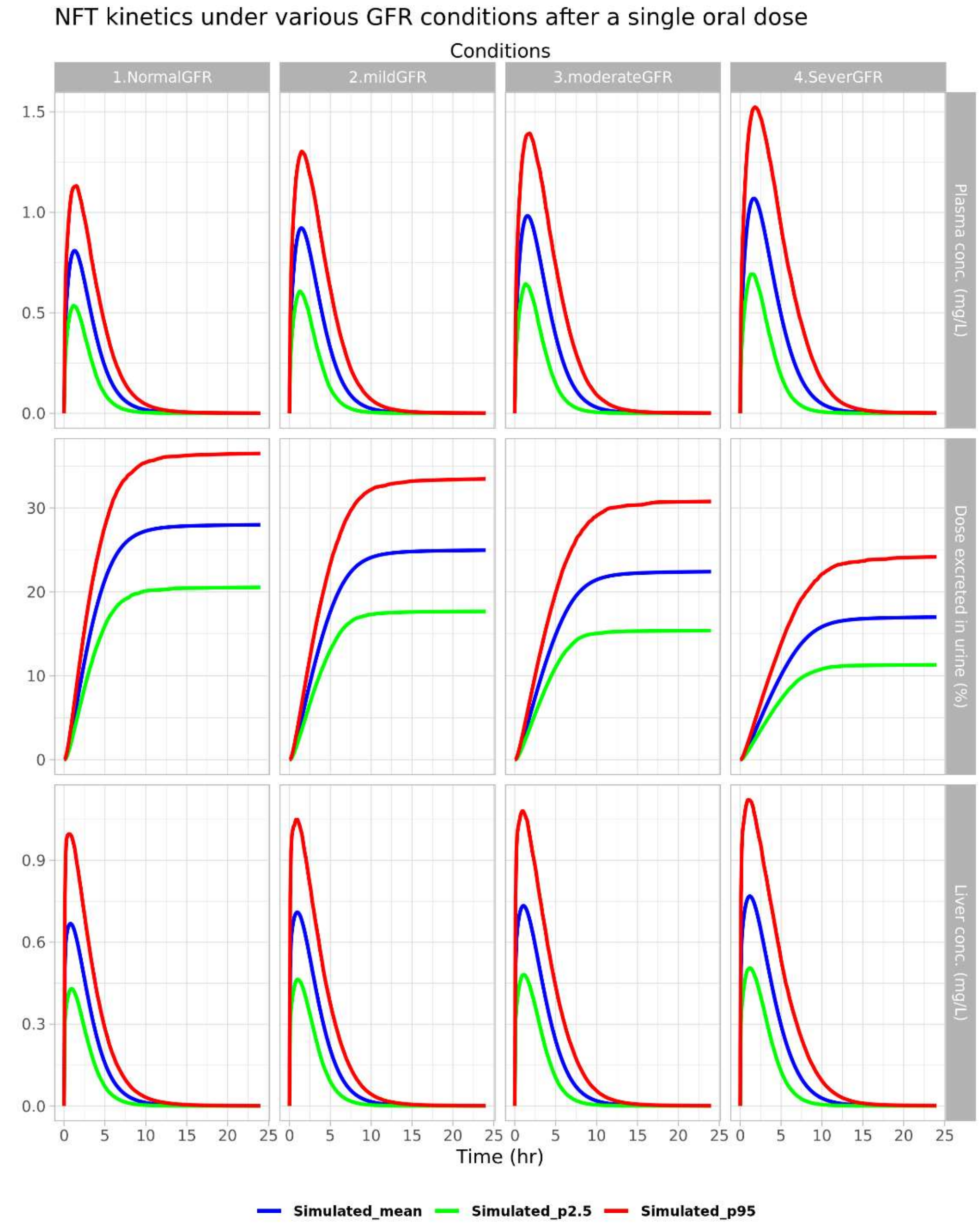

Figure S12. Predicted influence of GFR condition on NFT plasma, liver, and urine concentrations. The human PBPK model V5a was simulated with a single dose (100 mg) of orally administered NFT. Solid lines represent simulated 2.5th (green) and 97.5th (red) percentile and mean values (blue). The simulations were calculated based on 2000 parameter values randomly selected from a Gaussian distribution centered around the fitted mean value per parameter (with a standard deviation of 1.17 on a log scale). The corresponding organs are indicated at the right panel of each plot, with various GFR conditions indicated above the panels (normal: GFR > 90 mL/min, moderate: GFR = 70 mL/min, mild: GFR= 45 mL/min, Severe: GFR = 20 mL/min).

Table S1. Rat physiological parameters.

Parameter	Symbol	Value	Unit	References
Body weight	BW	0.25	kg	
Cardiac blood output	QCC	15.7	(L/hr/$kg^{.74}$)	(Brown et al., 1997)<br>(Davies and Morris, 1993)
Fractional liver blood flow	FQliver	0.174	-	(Campbell et al., 2016)
Fractional gut blood flow	FQgut	0.021	-	(Brown et al., 1997)
Fractional kidney blood flow	FQkidney	0.141	-	(Brown et al., 1997)
Fractional fat blood flow	FQfat	0.07	-	(Campbell et al., 2016)
Glomerular filtration rate (GFR)	Qfilterate	0.129	L/h	(Katayama et al., 2010)
	Constant Fraction of organ volume to body weight			
Fractional liver volume	Fliver	0.026	-	(Brown et al., 1997)
Fractional kidney volume	Fkidney	0.0073	-	(Brown et al., 1997)
Fractional tubules volume	Ffiltrate	0.00073	-	10 percent of Kidney volume
Fractional fat volume	Ffat	0.187	-	(Brown et al., 1997)
Fractional plasma volume	Fplasma	0.0428	-	(Davies and Morris, 1993)

Table S2. Rabbit physiological parameters.

Parameter	Symbol	Value	Unit	References
Body weight	BW	2.5	kg	
Cardiac blood output	QCC	15.96	L/hr/$kg^{.74}$	(Brown et al., 1997)<br>(Davies and Morris, 1993)
Fractional gut blood flow	FQgut	0. 209	-	(Brown et al., 1997) (Davies and Morris, 1993)
Fractional liver blood flow	FQliver	0.1245	-	(Brown et al., 1997) (Davies and Morris, 1993)
Fractional kidney blood flow	FQkidney	0.151	-	(Brown et al., 1997) (Davies and Morris, 1993)
Fractional fat blood flow	FQfat	0.06	-	(Brown et al., 1997)
Glomerular filtration rate (GFR)	Qfiltrate	0.62	L/hr	(Michigoshi et al., 2012)
Constant Fraction of organs volume to body weight				
Fractional gut volume	Fgut	0.048	-	(Brown et al., 1997) (Davies and Morris, 1993)
Fractional liver volume	Fliver	0.04	-	(Brown et al., 1997) (Davies and Morris, 1993)
Fractional kidney volume	Fkidney	0.006	-	(Brown et al., 1997) (Davies and Morris, 1993)
Fractional filtrate volume	Ffiltrate	0.0006	-	(Brown et al., 1997) (Davies and Morris, 1993)
Fractional fat volume	Ffat	0.048	-	(Brown et al., 1997) (Davies and Morris, 1993)
Fractional plasma volume	Fplasma	0.085	-	(Brown et al., 1997) (Davies and Morris, 1993)

Table S3. Human adult physiological parameters.

Parameter	Symbol	Value	Unit	References
Body weight	BW	70	kg	
Cardiac blood output	QCC	4.8	L/h/kg	(Brown et al., 1997) (Davies and Morris, 1993)
Fractional liver blood flow	FQliver	0.25	-	(Brown et al., 1997)
Fractional gut blood flow	FQlung	0.034	-	(Brown et al., 1997)
Fractional kidney blood flow	FQkidney	0.177	-	(Brown et al., 1997)
Fractional fat blood flow	FQfat	0.052	-	(Brown et al., 1997)
Glomerular filtration rate (GFR)	Qfiltrate	7.2	L/hr	
	Constant Fraction of organ volume to body weight			
Fractional liver volume	Fliver	0.026	-	(Brown et al., 1997)
Fractional kidney volume	Fkidney	0.0073	-	(Brown et al., 1997)
Fractional filtrate volume	Ffiltrate	0.00073	-	
Fractional fat volume	Ffat	0.187	-	(Brown et al., 1997)
Fractional plasma volume	Fplasma	0.0428	-	(Davies and Morris, 1993)
Fractional gut volume	Fgut	0.016	-	(Brown et al., 1997)

Table S4. PBPK biochemical parameters for three different species.

Parmeters	Symbol	Rabbit mean (min-max)	Rat mean (min-max)	Human mean (min-max)	Units	Type
Gut to plasma partition coefficient	Kgut_plasma	0.622			-	Calculated
Liver to plasma partition coefficient	Kliver_plasma	0.651			-	Calculated
Kidney to plasma partition coefficient	Kkidney_plasma	0.671			-	Calculated
Fat to plasma partition coefficient	Kfat_plasma	0.159			-	Calculated
Liver to plasma partition coefficient	Krestbody_plasma	0.423 (0.39-0.45)			-	Fitted
Fraction unbound to plasma	fu	0.42			-	(Watari et al., 1985)
Urine excretion rate	QurineC*	11.45 (7.92-20.24)			$hr^{-1}kg^{-1}$	Fitted
Tubular reabsorption rate	krc*	1.33 (1.11-1.56)			mg/l/hr/kg	Fitted
Maximum active tubular secretion	Tmc*	8.02 (6.78-9.30)			mg/hr/kg	Fitted
Half maximal concentration of active tubular secretion	Kt	0.059 (0.043-0.079)			mg/l	Fitted
Feces excretion rate	kfeces	3.34 (0.23 - 7.46)	0.0187 (0.00063 - 0.0649)		hr^{-1}	Fitted
Maximum rate of NFT metabolism in liver	VmaxC*	0.47 (0.42-0.53)			mg/hr/kg	Fitted
Half maximal concentration of NFT metabolism in liver	Km	5.83 (4.96-6.82)			mg/l	Fitted
Maximum rate of NFT hepatobiliary excretion	Vehrc**	0.022 (0.0012 - 0.104)	0.52 (0.43-0.66)		mg/hr/kg	Fitted

Half maximal concentration of NFT hepatobiliary excretion	Kehr**	3.69 (0.74 -7.81)	0.017 (0.0014-0.063)	mg/l	Fitted
Rate at which NFT transfer back to gut from bile	kbile**	3.36 (0.2-8.98)	0.256 (0.007- 0.83)	$hr^{-1}kg^{-1}$	Fitted
Absorption rate constant	kgutabs**	0.30 (0.07-0.66)	2.11 (1.80 -2.48)	$hr^{-1}kg^{-1}$	Fitted
Blood to plasma partition coefficient	Kbp	0.76		-	(Zhang et al., 2022)

Note: To scale the value to human we used allometric scaling with the equation below. Parameters with a single star (*) use rabbit body weight and with double stars (**) use rat bodyweight as a basis for allometric scaling to human.

$$Parameter_{human} = Parameter_{species} * \left(\frac{BW_{species}}{BW_{human}}\right)^{0.25}$$

Standard ordinary differential equations used in PBPK model for NFT

$$\frac{d}{dt}A_{gutlumen} = -kgutabs * A_{gutlumen} - kfeces * Agutlumen + kbile * Abile + input$$

- $A_{gutlumen}$ is the amount of NFT in the gut lumen
- $kgutabs$ is NFT transfer rate of the gut lumen to gut
- $kfeces$ is the rate at which NFT excretes into feces
- $kbile$ is the rate at which NFT transfers from bile to gut
- $Abile$ is the amount of NFT in bile
- $input$ is the amount corresponding to the administered dose

$$\frac{d}{dt}(A_{gut}) = kgutabs * A_{gutlumen} + Qgut * \left(cplasma * \left(\frac{fu}{Kbp}\right) - cgut * \left(\frac{\frac{fu}{Kbp}}{Kgut_{plasma}}\right)\right)$$

- $Agut$ is the amount of NFT in the gut
- $Qgut$ is the blood flow to the gut
- $cgut$ is the concentration of NFT in the gut
- $cplasma$ is the plasma concentration of NFT
- fu is the free fraction and is the same for every tissue
- Kbp is the blood plasma partition coefficient and is the same for every tissue
- $Kgut_{plasma}$ is the gut to plasma partition coefficient

$$\frac{d}{dt}(ALiver) = Qliver * cplasma * \left(\frac{fu}{Kbp}\right) + Qgut * cgut * \left(\frac{\frac{fu}{Kbp}}{Kgut_{plasma}}\right) - (Qliver + Qgut) * cliver * \left(\frac{\frac{fu}{Kbp}}{Kliver_{plasma}}\right) - \frac{Vmax * cliver * fu}{Km + cliver * fu} - \frac{Vehr * cliver * fu}{Kehr + cliver * fu}$$

- $ALiver$ is the amount of NFT in the liver
- $Qliver$ is the cardiac blood flow to liver
- $cliver$ is the concentration of NFT in the liver
- $Kliver_{plasma}$ is the liver plasma partition coefficient
- $Vmax$ is the maximal NFT metabolism rate
- Km is the concentration at which NFT metabolism is half-maximal
- $Vehr$ is the maximum rate of hepatobiliary excretion
- $Kehr$ is the concentration at which hepatobiliary excretion is half-maximal

$$\frac{d}{dt}(ABile) = \frac{Vehr * cliver * (fu)}{Kehr + cliver * (fu)} - kbile * ABile$$

$$\frac{d}{dt}(Afat) = Qfat * cplasma * (fu/Kbp) - Qfat * cfat * \left(\frac{\frac{fu}{Kbp}}{Kfat_{plasma}}\right)$$

- $Afat$ is the amount of NFT in fat
- $Qfat$ is the blood flow to fat
- $Kfat_{plasma}$ fat plasma partition coefficient

$$\frac{d}{dt}(Akidney) = Qkidney * cplasma * \left(\frac{fu}{Kbp}\right) - Qkidney * ckidney * \left(\frac{\frac{fu}{Kbp}}{Kkidney_{plasma}}\right) - Qfiltrate * ckidney * kidney * \left(\frac{\frac{fu}{Kbp}}{Kkidney_{plasma}}\right) - \frac{Tm * ctubules}{Kt + ctubules} + kr * ctubules$$

- $Akidney$ is the amount of NFT in the kidney
- $Qkidney$ is the blood flow to kidney
- $Kkidney_{plasma}$ is the kidney plasma partition coefficient
- $Qfiltrate$ is the glomerular filtration rate
- kr is the tubular reabsorption rate
- Tm is maximal active tubular secretion rate of NFT
- Kt is the concentration at which NFT secretion is half-maximal
- $ctubules$ is the NFT concentration in tubules

$$\frac{d}{dt}(Atubules) = Qfiltrate * ckidney * kidney * \left(\frac{\frac{fu}{Kbp}}{Kkidney_{plasma}}\right) - Qfiltrate * ctubules - Tr * ctubules + \frac{Tm * ctubules}{Kt + ctubules}$$

- $Afiltrate$ is the amount of NFT in tubules

$$\frac{d}{dt}(Adelay) = Qfiltrate * ctubules - Qurine * Adelay$$

- $Adelay$ is the amount of NFT in the delay compartment (urine storage)
- $Qurine$ is the rate at which NFT transfers to urine

$$\frac{d}{dt}(Aurine) = Qurine * Adelay$$

- $Aurine$ is the amount of NFT in the urine storage
- $Qurine$ is the rate at which NFT transfers to urine

$$\frac{d}{dt}(Arestbody) = Qrestbody * cplasma * \left(\frac{fu}{Kbp}\right) - Qrestbody * crestbody * \left(\frac{\frac{fu}{Kbp}}{Krestbody_{plasma}}\right)$$

- $Arestbody$ is the amount of NFT in the rest of the body
- $Qrestbody$ is the blood flow to the rest of the body
- $Krestbody_{plasma}$ is the restbody plasma partition coefficient

$$\frac{d}{dt}(Aplasma) = (Qliver + Qgut) * cliver * \left(\frac{\frac{fu}{Kbp}}{Kliver_{plasma}}\right) + Qkidney * ckidney * \left(\frac{\frac{fu}{Kbp}}{Kkidney_{plasma}}\right) + Qfat * cfat * \left(\frac{\frac{fu}{Kbp}}{Kfat_{plasma}}\right) + Qrestbody * crestbody * \left(\frac{\frac{fu}{Kbp}}{Krestbody_{plasma}}\right) - QCplasma * cplasma * \left(\frac{fu}{Kbp}\right)$$

- $Aplasma$ is the amount of NFT in plasma
- $QCplasma$ is the cardiac output for the plasma flow

References:

Brown, R.P., Delp, M.D., Lindstedt, S.L., Rhomberg, L.R., Beliles, R.P., 1997. Physiological parameter values for physiologically based pharmacokinetic models. Toxicol. Ind. Health 13, 407–484.

Campbell, J.L., Andersen, M.E., Hinderliter, P.M., Yi, K.D., Pastoor, T.P., Breckenridge, C.B., Clewel, H.J., 2016. PBPK model for atrazine and its chlorotriazine metabolites in rat and human. Toxicol. Sci. 150, 441–453. https://doi.org/10.1093/toxsci/kfw014

Davies, B., Morris, T., 1993. Physiological Parameters in Laboratory Animals and Humans. Pharm. Res. 10, 1093–1095. https://doi.org/10.1023/A:1018943613122

Katayama, R., Yamaguchi, N., Yamashita, T., Watanabe, S., Satoh, H., Yamagishi, N., Furuhama, K., 2010. Calculation of glomerular filtration rate in conscious rats by the use of a bolus injection of iodixanol and a single blood sample. J. Pharmacol. Toxicol. Methods 61, 59–64. https://doi.org/10.1016/j.vascn.2009.10.002

Michigoshi, Y., Katayama, R., Yamagishi, N., Kato, M., Saito, J., Satoh, H., Furuhama, K., 2012. Estimation of glomerular filtration rate in rabbits by a single-sample method using iodixanol. Lab. Anim. 46, 341–344. https://doi.org/10.1258/la.2012.011065

Watari, N., Aizawa, K., Kaneniwa, N., 1985. Dose- and time-dependent kinetics of the renal excretion of nitrofurantoin in the rabbit. J. Pharm. Sci. 74, 165–170. https://doi.org/10.1002/jps.2600740212

Zhang, T., Zou, P., Fang, Y., Li, Y., 2022. Physiologically based pharmacokinetic model to predict drug concentrations of breast cancer resistance protein substrates in milk. Biopharm. Drug Dispos. 43, 221–232. https://doi.org/10.1002/bdd.2335
